# Crystal structure and biophysical characterisation of the enterococcal foldase PpiC, a cross‐opsonic antigen against gram‐positive nosocomial pathogens

**DOI:** 10.1111/febs.70160

**Published:** 2025-06-30

**Authors:** Valeria Napolitano, Eliza Kramarska, Ornella Ghilardi, Felipe Romero‐Saavedra, Pompea Del Vecchio, Flavia Squeglia, Johannes Huebner, Rita Berisio

**Affiliations:** ^1^ Institute of Biostructures and Bioimaging, Italian Research Council (CNR) Naples Italy; ^2^ Łukasiewicz Research Network – PORT Polish Center for Technology Development Wroclaw Poland; ^3^ Division of Paediatric Infectious Disease, Hauner Children's Hospital LMU Munich Germany; ^4^ Department of Chemical Sciences University of Naples Federico II Italy

**Keywords:** foldase, gram‐positive, infectious disease, protein folding, protein structure

## Abstract

*Enterococcus faecium* have high rates of antibiotic resistances, with vancomycin‐resistant *E*. *faecium* acknowledged as the most important in the clinical setting and declared by WHO to be a threat to humankind, for which rapid actions are needed. PpiC is a membrane‐bound lipoprotein of *E*. *faecium* endowed with both a peptidyl‐prolyl isomerase and a foldase activity, and plays a key role in assisting the folding of many secreted enterococcal proteins. It is located at the membrane–wall interface, therefore easily accessible to inhibitors and to the immune system and an ideal target for drug and vaccine development. Despite their potential, enterococcal peptidyl‐prolyl isomerases have been understudied. We previously identified PpiC as an important cross‐protective vaccine antigen. To gain a better understanding of the PpiC biological role in *E*. *faecium* survival, we determined the crystal structure of PpiC and investigated its biophysical properties. Consistent with PpiC's folding activity, the biological assembly of PpiC is a bowl‐shaped structure containing two parvulin‐type peptidyl‐prolyl cis/trans isomerase domains. We also dissected the role of *N*‐ and *C*‐terminal regions of the molecule in its dimerisation, an event which is predicted to play an important role in the folding of client proteins. Our data point to a functional cross‐talk between the foldase and peptidyl‐prolyl isomerase activities of PpiC, through the protein‐swapping involved in dimerisation. Also, our work provides key structural data for the design of antimicrobials and cross‐protective vaccine antigens against nosocomial infections.

## Graphical abstract

PpiC is an enterococcal lipoprotein endowed with both Peptidyl‐prolyl cis‐trans isomerase (PPIase) and foldase activities. It is a potential target for novel antimicrobials and a promising vaccine antigen. We determined the crystal structure of the enzyme, showing that PpiC adopts a dimeric organisation embedding two catalytic parvulin‐type PPIase domains joined by a large crevice. Biophysical studies suggested the importance of protein swapping in the PpiC dimerisation and folding.

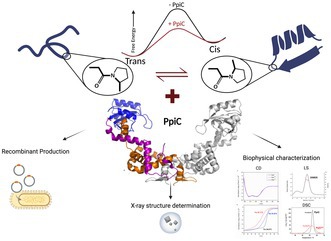

**Abbreviations**
CD: circular dichroism
DSC: differential scanning calorimetry
EP: electrostatic potential
LS: light scattering
MW: molecular weight
NC domain: PPIase foldase domain formed by *N*‐ and *C*‐terminal regions
NC: foldase domain of PPIases
PBP: penicillin‐binding protein
PDB: Protein Databank
PFAM: Protein Family Database
PPIase: peptidyl‐prolyl isomerase
PpiC: peptidyl‐prolyl isomerase C
RMSD: root mean square deviation
SEC‐LS: size exclusion chromatography coupled with light scattering
Tm: melting temperature
UV‐CD: Ultraviolet Circular Dichroism
VRE: vancomycin‐resistant *Enterococcus faecium*
WHO: World Health Organisation

## Introduction

*Enterococci* are ubiquitous gram‐positive, facultative anaerobic microorganisms, which colonise a broad range of hosts, from invertebrates to mammals, including humans [1, 2]. It was shown in mice that *enterococci* can invade extra‐intestinal regions, in a process facilitated by the administration of antibiotics [3, 4]. Therefore, *E*. *faecium* and *E*. *faecalis* [5, 6] are the major etiological agents of nosocomial infections as they infect patients with recent surgery, organ transplantation, diabetes, malignancy, and renal insufficiency [7, 8]. Although both strains have clinical importance, *E*. *faecium* infections have higher rates of antibiotic resistance and mortality [9]. *Enterococci* often have intrinsic tolerance and/or resistances to many antibiotics (e.g. beta‐lactams [10, 11, 12, 13], cephalosporins [14, 15], glycopeptides, aminoglycosides [16, 17]), and acquired resistances toward gentamicin and streptomycin [18, 19]. Moreover, vancomycin resistance is important in the clinical setting because of its use for other MDR gram‐positive bacteria, and for patients allergic to semi‐synthetic penicillins and cephalosporins [20]. In 2017, the WHO declared vancomycin‐resistant *E*. *faecium* (VRE) a threat to humankind for which rapid actions are needed [21, 22].

The folding and maturation of many secreted enterococcal proteins depend on an extracellular foldase, a lipoprotein endowed with both Peptidyl‐prolyl cis‐trans isomerase (PPIase) and foldase activities [23]. Indeed, cis‐trans isomerisation is the rate‐limiting step in the folding of proteins containing cis‐prolines [24]. In *E*. *faecalis*, the PPIase denoted as PrsA was found to be a potential virulence factor, mediating high resistance to salt concentrations and ampicillin. The protein was also involved in the folding and trafficking of penicillin‐binding proteins (PBPs) [25]. PrsA was also discovered in *Bacillus subtilis* and other Firmicutes [26]. Most recently, the PPIases PrsA and SlrA of *Streptococcus pneumoniae* were shown to play an important role in the secretion of the cholesterol‐dependent pore‐forming toxin pneumolysin [27].

Some PPIases act as selective enzymes and recognise proline residues preceded only by certain signals, such as phosphorylated amino acids as in the case of human Pin1 [28, 29, 30]. Other PPIases can act on a wide range of substrates, and recognition can be phosphorylation‐independent [25]. Within PPIases, we can distinguish three families: the cyclophilins, the FK506 binding proteins, and the parvulins [31, 32]. At their *N*‐terminus, PPIases typically present a signal sequence followed by a flexible linker containing a cysteine residue that is enzymatically modified upon signal sequence cleavage with a diacylglycerol residue for membrane association. Parvulin‐type PPIases contain a family‐conserved core region, generally embedding a parvulin domain and a chaperone domain composed of both *N*‐ and *C*‐terminal regions. To date, it is unclear whether the chaperone and parvulin domains are in an inter‐domain cross‐talk, that is if the chaperone domain is actively delivering substrates to the active site of the parvulin domain or the two functions are completely independent.

Due to the importance of their catalysed reaction, PPIases are potential targets for novel antimicrobials. However, despite the clinical relevance of *enterococci*, their PPIases have been poorly investigated. We previously identified the PPIase PpiC of *E*. *faecium* as a promising vaccine antigen, as it can induce the production of opsonic and cross‐protective antibodies targeting *enterococci* [33]. Also, we proved that rabbit serum raised against PpiC can mediate opsonophagocytic killing of several *S. aureus* strains (i.e. MW2, LAC, and Reynolds) [34], suggesting that PpiC can act as a cross‐reactive antigen, similar to other recently discovered antigens [35]. Given the relevance of PpiC as a drug target and as a promising antigen against multi‐resistant gram‐positive pathogens, we decided to investigate its structural features. Here, we determined the crystal structure of the enzyme at 2.5 Å resolution, showing that PpiC adopts a dimeric organisation embedding two catalytic parvulin‐type PPIase domains joined by a large crevice. This crevice is likely functional as an extended interactive surface to help the folding of client proteins. Also, we set out to investigate the structural determinants of PpiC oligomeric organisation, an important feature in PPIases for their interactions with client proteins [36]. Biophysical studies of truncated forms of the enzyme suggested the existence of a cross‐talk between parvulin and chaperone domains of PpiC, that allows it to fold cooperatively. This cross‐talk, mediated by the swapping of *N*‐ and *C*‐terminal regions, is likely responsible for the concerted action of the two catalytic functions of PpiC. Finally, our data provide a structural basis for the structure‐based design of improved vaccine antigens and for the design of specific inhibitors.

## Results

### PpiC is a stable and dimeric protein

**Fig. 1:**
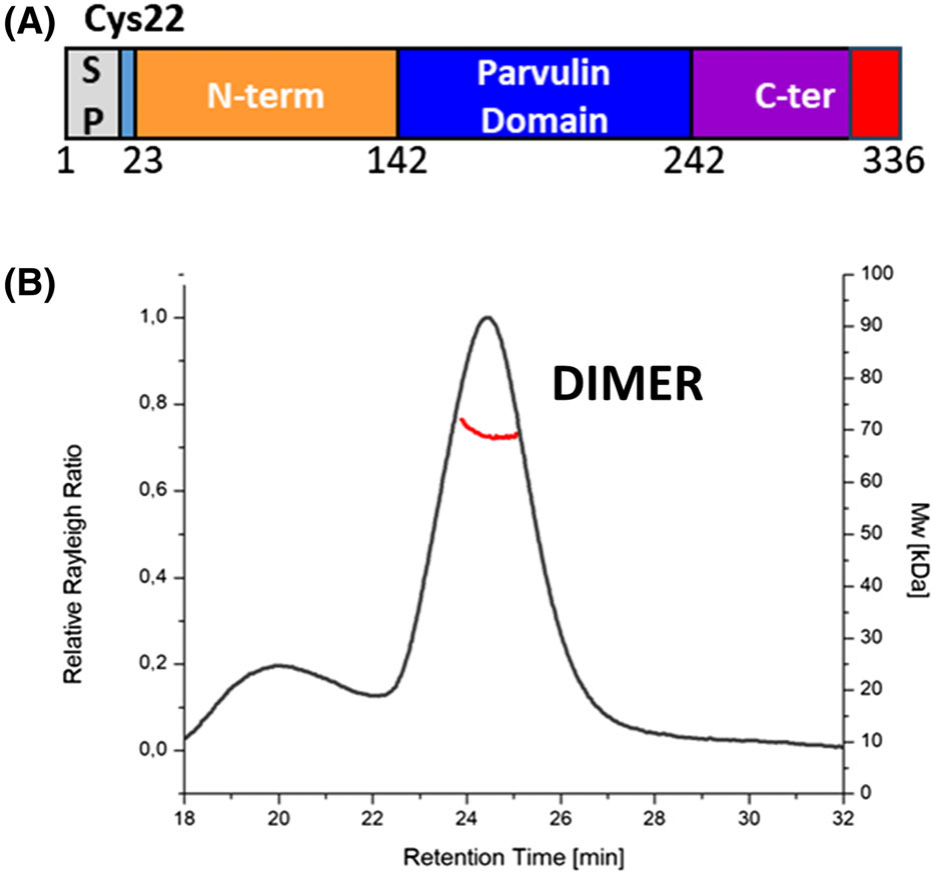
PpiC is a multi‐domain dimeric protein. (A) Domain boundaries in PpiC sequence; *N*‐terminus, parvulin and *C*‐terminus domain are reported in yellow, blue and purple, respectively. The signal peptide at the *N*‐terminal ends and the flexible *C*‐terminus (residues 331–336) is coloured in grey and red, respectively. (B) Analytical size exclusion chromatography coupled with light‐scattering (SEC‐LS) of PpiC. Relative Rayleigh ratios (left scale) and derived molar masses (right scale, red points) are plotted versus elution time.

Native PpiC of *E*. *faecium* is a 336 residues protein belonging to the PrsA family of foldases, as predicted through searches in the Protein Family Database (PFAM) database [37]. From its *N*‐terminal side, the protein embeds a signal peptide, followed by a linker region including Cys22. In other PPIases, cleavage occurs upstream from the cysteine, which is enzymatically modified to accommodate the cell wall‐anchoring lipo‐box (diacylglycerol) [38]. According to the PFAM database [37], the mature enzyme starts with Cys22, and contains a trigger factor/SurA domain superfamily and a peptidyl‐prolyl cis‐trans isomerase (PPIase) domain between residues 142–223 (Fig. 1A). PPIase domains are known to accelerate protein folding by catalysing the cis‐trans isomerisation of proline imidic peptide bonds in oligopeptides [38]. The protein terminates with a strongly negative *C*‐terminal end, rich in Asp and Glu amino acids (304–336).

To experimentally assess the structural and unfolding features of PpiC of *E*. *faecium*, we recombinantly produced and purified PpiC depleted of the signal peptide (residues 1–26) in high yields. As shown in Fig. 1B, light‐scattering experiments unambiguously show that PpiC has a dimeric structure. Indeed, intensity of the Rayleigh scattering provides a weight average MW of 69.29 ± 0.99 kDa (Fig. 1B). Dimerisation is often observed in foldases, as it leads to a large crevice that may protect substrate proteins from aggregation *in vivo* [39].

### Crystal structure of PpiC dimer

The purified PpiC of *E*. *faecium* was crystallised in the monoclinic space group P2_1_ with cell dimensions of *a* = 67.5, *b* = 69.7, *c* = 87.4 Å, *β* = 102.8°, and two protein molecules in the asymmetric unit. The crystals diffracted beyond 2.5 Å resolution, and the structure was solved by the molecular replacement method, using Phaser [40]. The best search model was obtained using the alphafold 3.0 tool, which produced a reliable monomeric structure for the region T30–T300 (PlDDt > 90) [41]. The molecular replacement procedure identified two molecules in the asymmetric unit. After several rounds of model building and refinement, the final model includes residues 29–303 (chain A) and 30–300 (chain B), 15 Cd^2+^ ions and 109 water molecules (Table 1). No signs of electron density were visible for the *C*‐terminal charged arm (residues 304–336). Consistently, this region is rich in serine and threonine residues and acidic residues (301‐SAFTTTSSSTKDSSETTASTKSSDTKSTDSTKESSTEETTDSSK‐336), with an isoelectric point of 4.5, and is predicted to be fully flexible [42]. Statistics of data collection and refinement are reported in Table 1.

**Table 1:** Data collection and refinement statistics. Values in parentheses are for the highest resolution shell, 2.6–2.5 Å.

*Data collection*	
Wavelength	0.8731
Beamline	ESRF beamline ID30B
Detector	DECTRIS EIGER X 4 M
Oscillation step	0.1°
Number of fremes	3600
*Data processing*	
Space group	P2_1_
Unit‐cell parameters *a*, *b*, *c*; β	67.5, 69.7, 87.4 Å; β = 102.8°
Resolution range (Å)	65.8–2.5
Total no. of reflections	50 297 (3103)
No. of unique reflections	17 572 (879)
Completeness (%)	86.7 (63.2)
* *R*_merge_ (%)	21.7 (147)
Average *I*/*σ*(*I*)	5.1 (1.5)
CC1/2	88.7 (50.1)
*Refinement*	
* *R*work/*R*free (%)	21.2/24.9
No. of residues	546
No. of water molecules	109
RMS deviations	
Bond lengths (Å)	0.003
Bond angles (°)	0.79
MolProbity score	2.93
MolProbity clashscore	14.5
Ramachandran favoured (%)	90.6
Ramachandran outliers (%)	1.7
Average B‐factor	47.5
Chain A	
All atoms	50.4
Main chain	48.6
Side chain	51.3
Chain B	
All atoms	44.5
Main chain	42.0
Side chain	45.8
Solvent	33.3

* *R*_merge_ = ∑ *h*∑i |*I*(*h*,*i*)– < *I*(*h*) > |/∑ h∑i *I*(*h*,*i*), where *I*(*h*,*i*) is the intensity of the *i*th measurement of reflection *h* and < *I*(*h*) > is the mean value of the intensity of reflection *h*.

**Fig. 2:**
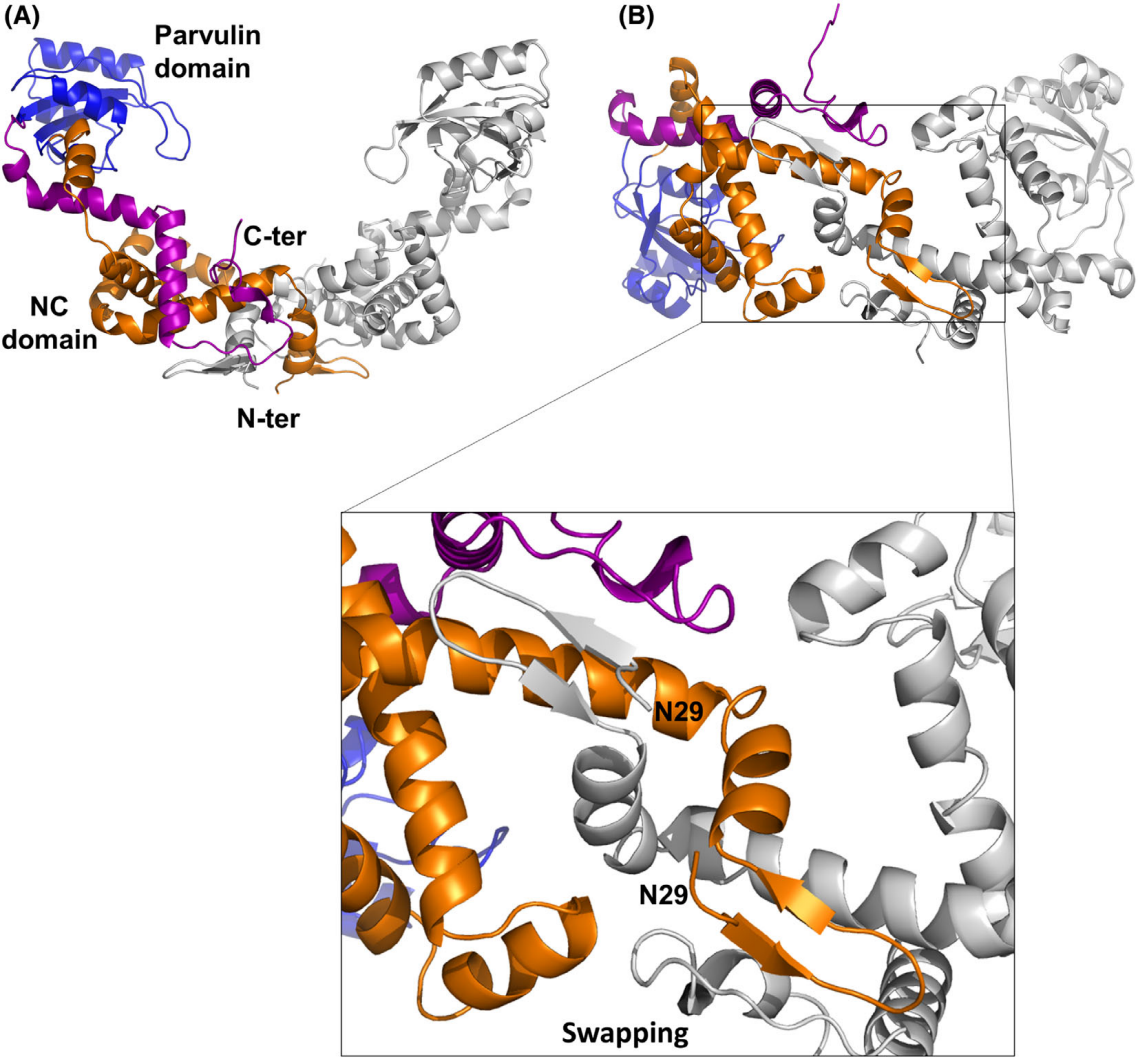
Cartoon representation of PpiC crystal structure. In the side (A) and bottom views (B), chain B is drawn in grey whereas in chain A the parvulin and the foldase (NC) domains are colour coded. The parvulin domain (residues 144–243) is coloured blue. The NC domain is composed of *N*‐terminal residues 51–143 (drawn in orange), and the *C*‐terminal residues 244–301 (drawn in purple) and is completed by the swapping of the *N*‐terminal residues 29–50 of the adjacent chain (grey). A zoom of the swapping region is reported as an inset of panel B. The figure was generated using pymol1.1.

The structure of PpiC presents a highly symmetric bowl‐shaped dimer, with root mean square deviations (RMSD) computed on backbone atoms of the two chains of 0.5 Å (Fig. 2A,B). Each chain of PpiC is formed by two domains, the catalytic parvulin‐type PPIase domain and a domain formed by both an *N*‐terminal and *C*‐terminal region, here denominated as the NC domain (Fig. 2). The NC domain, also known as foldase domain, is formed by three different regions. A swapped *N*‐terminal β‐hairpin‐helix motif (residues 29–50) belonging to one chain interacts with both an *N*‐terminal (residues 51–143) and a *C*‐terminal region (redidues 244–301) of the adjacent chain (Fig. 2).

**Fig. 3:**
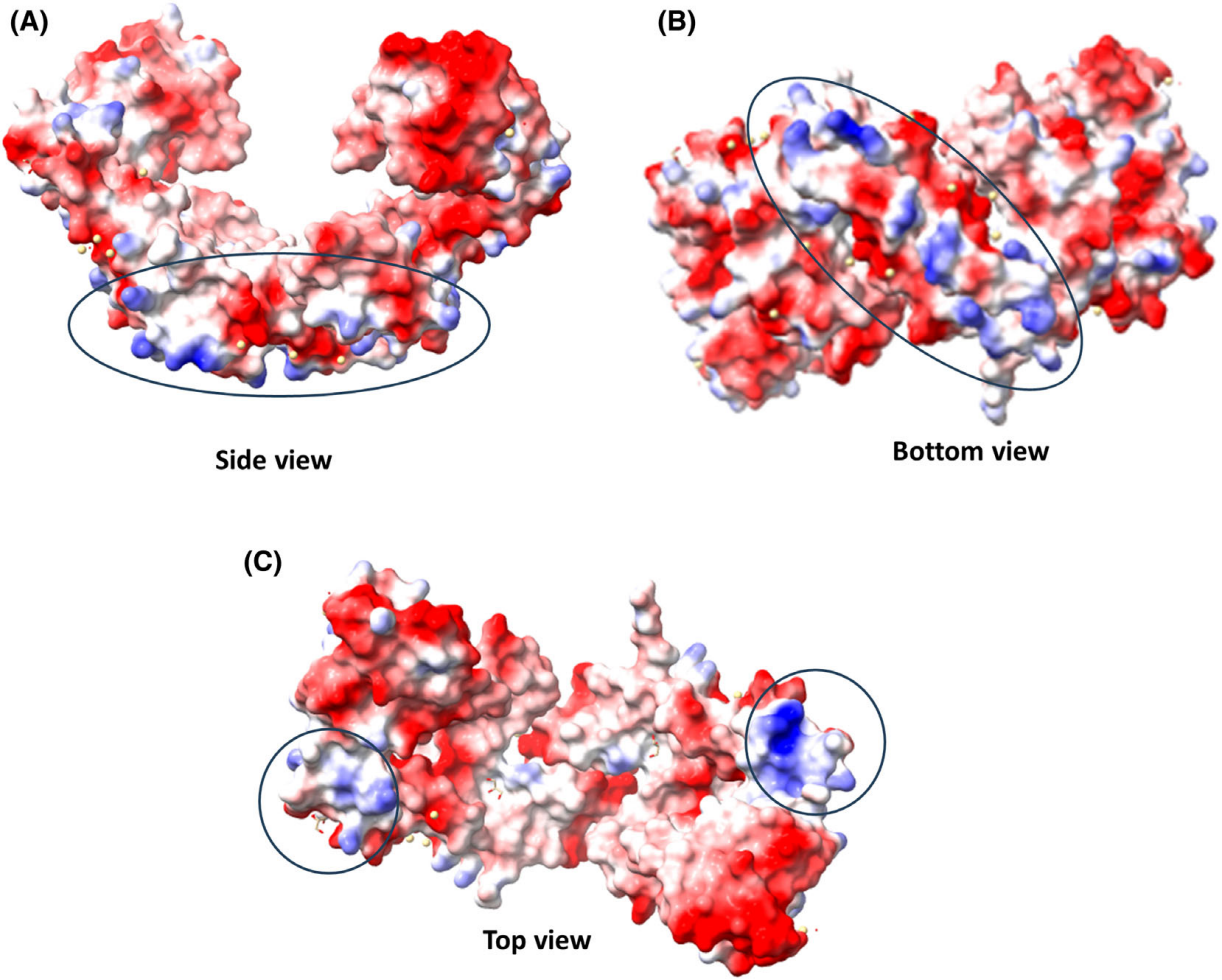
Electrostatic potential (EP) surface of PpiC. (A–C) Three views of EP (residues 27–303), computed with chimerax, are shown. The black circles highlight the positively charged EP surface areas.

The swapped *N*‐terminal β‐hairpin regions, located close to the lipo‐box carrying Cys22, point toward the *E. faecium* membrane surface and potentially interact with it. Consistently, the analysis of the electrostatic potential surface of the PpiC structure, computed using ChimeraX [43], shows that PpiC is an almost fully negatively charged molecule, with the most prominent positively charged region in the bottom part of the molecule, constituted by the swapped β‐hairpin regions (Fig. 3A,B). Another positively charged region is a lysine‐rich region connecting the NC domain with the parvulin domain of each chain (residues 238–250) (Fig. 3C). Although PpiC substrate client proteins are hitherto unknown, it is well established that electrostatic interactions provide the initial driving force for attraction of foldases to the unfolded client [44, 45]. Therefore, it can be assumed that PpiC is endowed with a specific preference for positively charged proteins.

**Fig. 4:**
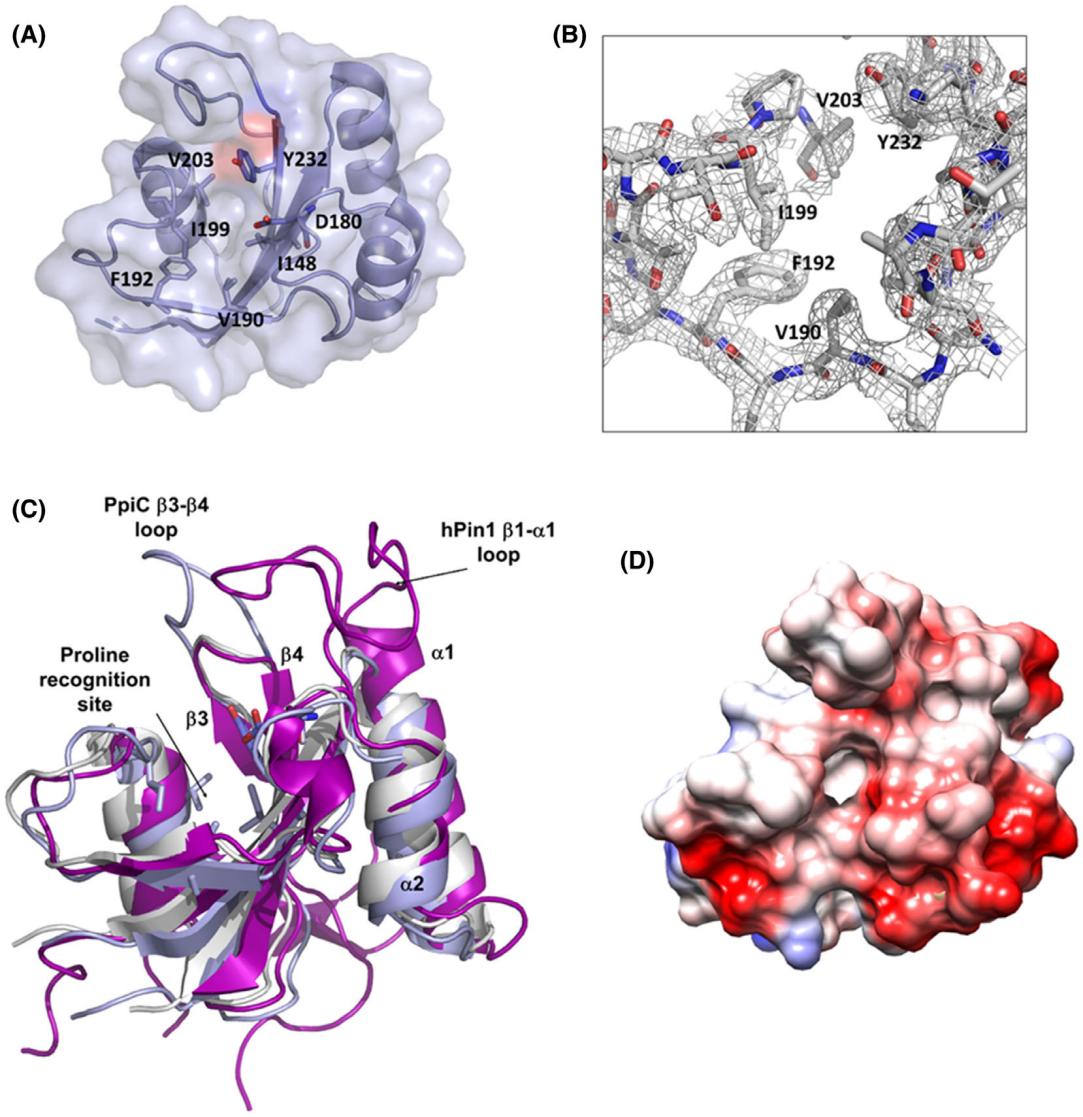
Structural features of PpiC catalytic domain. (A) Cartoon and surface representation of PpiC parvulin domain. Residues lining the catalytic pocket are drawn in sticks. (B) (2Fo–Fc) electron density map contoured at 2*σ*, at PpiC catalytic cleft. (C) Cartoon representation of PpiC parvulin domain (light blue) superposed to hPin1(purple) and PrsA of *B. subtilis* (white). (D) Electrostatic potential surface of PpiC parvulin domain. The figure was generated using pymol1.1.

Five homologous proteins were detected in the PDB database using the tool blastp [46] (Table 2), including PPIases from *S. pneumoniae*, *S*. *mutans*, *L. monocytes* and *B. subtilis* [27, 36, 39] (Table 2). Despite the general overall conserved fold with these five proteins, we observe large structural variations among them, mainly in the relative orientations of the PPIase domains with respect to the NC domains. Consistently, backbone RMSD values computed upon superposition of each of them on the structure of PpiC are in a wide range, from 2.3 Å in the case of *S. pneumoniae*, to 8.6 Å in the case of *B. subtilis* (Table 2). After superposition of NC domains, rotational angles of the PPIase domain range from 8.3° in the case of *S. pneumoniae* to 70° in the case of *B. subtilis* (Table 2, Fig. 4A). A high flexibility is a typical feature of foldases, as flexibility allows the client proteins to sample a wide conformational space while still bound to the chaperone [44].

**Table 2:** PpiC homologous structures in the Protein Databank.

Description	Scientific name	Query cover (%)	Sequence identity (%)	Accession	Backbone RMSD upon superposition of PrsA chains with PpiC	Rotation of the PPiase domain after superposition of the NC domain (°)	References
PrsA	*Streptococcus mutans*	81	46.3	7L75_A	Chains A, B: 3.4, 3.5 Å	15.4	[27]
PrsA	*Streptococcus pneumoniae* str. Canada MDR_19A	78	41.1	5TVL_A	Chains A, B: 2.3, 3.2, 3.3, 4.0 Å	8.3	[27]
PrsA	*Lactococcus lactis* subsp. *lactis* Il1403	79	38.2	6VJ2_A	Chains A, B: 3.0, 3.5 Å	17.0	Not available
PrsA 1	*Listeria monocytogenes* EGD‐e	75	31.0	5HTF_A	Chains A, B: 4.5, 4.9 Å	28.3	[36]
PrsA	*Bacillus subtilis* subsp. *subtilis* str. 168	80	29.5	4WO7_A	Chains A, B: 7.2, 8.6 Å	70.0	[39]

### The catalytic PPIase domain

Typical of parvulin‐type PPIase domains, the parvulin domain of PpiC consists of a four‐stranded antiparallel β‐sheet surrounded by four α‐helices (E147‐N242) [47] (Fig. 4A,B). When compared to other known PPIase domains, an overall conservation of its structure is observed, with RMSD values computed on backbone atoms in the range 0.8–1.8 Å and sequence identities ranging between 29.5 and 46.3%. Structural similarity is also observed with the PPIase domain of human Pin1 (RMSD 1.3 Å), despite the low sequence identity (18%) [48]. Due to its relevance in cancer in activating oncogenes and inactivating tumour suppressors, the structure and function of hPin1 has been extensively studied [49, 50].

On analogy to PrsA and hPin1, a hydrophobic pocket is formed in PpiC by Ile199, Val203, and Val190, which likely constitutes the binding site for the cyclic side chain of proline (Fig. 4A). Indeed, the hydrophobic character of these residues is fully conserved in all homologous PPIases (Table 3). In hPin1, the peptidyl‐prolyl bond undergoing catalysed isomerisation is surrounded by side chains of residues Cys113, His59, His157, and Ser154 (corresponding to Asp180, Ile248, Tyr232 and Asn223 in PpiC, Table 3). As shown in Table 3, Cys113 of hPin1 is replaced by an aspartic residue in almost all bacterial PPIases, whereas His59 is conserved in several bacterial PPIases including PrsA of *B. subtilis* and *L. monocytes*. Conversely, a hydrophobic residue replaces His59 in PpiC (Ile248) and PrsA PPIases of *S. mutans* (Val148), *S. pneumoniae* (Ile150) and *L. lactis* (Val158) (Table 3). As for His157 and Ser154 of hPin1, their hydrogen‐bonding donor nature is almost fully conserved in all species (Tyr232 and Asn223 in PpiC, Table 3).

**Table 3:** Putative PpiC catalytic residues and corresponding residues in other PPIases.

*Enterococcus faecium* PpiC	Ile148	Asp180	Val190	Phe192	Ile199	Val203	Thr221	Asn223	Tyr232
*Streptococcus mutans* PrsA	Val151	Gly184	Tyr189	Phe191	Leu198	Val202	Ala220	Asp222	Tyr231
*Streptococcus pneumoniae* PrsA	Ile150	Asp183	Ile193	Phe195	Val202	Val206	Ala224	Gly226	Tyr235
*Lactococcus lactis* Foldase	Val158	–*	Val193	Phe195	Val202	Val206	Ser224	Ser226	Tyr235
*Listeria monocytogenes* PrsA 1	His142	Asp174	Leu184	Phe187	Met192	Phe196	Ser215	–*	His220
*Baciullus subtilis* PrsA	His123	Asp155	Leu164	Phe167	Met173	Phe177	Thr195	Tyr197	His200
*Homo sapiens* Pin1	His59	Cys113	Leu122	Phe125	Met130	Phe134	Thr152	Ser154	His157
*Escherichia coli* SurA‐P2	His178	Asp222	Leu341	Ala344	Leu239	Phe243	Ser261	Val263	His266
*Escherichia coli* Par10	His8	Cys40	Leu49	Phe52	Met57	Phe61	Thr79	Phe81	His84

* Not present in the specific structure due to loop deletion.

Compared to PpiC and PrsA, hPin1 contains a long positively charged loop, between β1 and α1, which forms the recognition site for the phosphorylated Ser/Thr‐Pro substrates [48]. This loop is replaced by a two‐residue turn in PpiC and in PrsA of *B. subtilis* and *L. monocytes* (Fig. 4C). On the other hand, a longer, albeit neutral loop, exists in PpiC between the β strands β3 and β4 (Fig. 4C), that is a two‐residue turn in both hPin1 and the PrsA PPIases of *L. monocytes* and *B. subtilis* (Fig. 4C). These findings suggest that, similar to PrsA proteins [39, 51], substrate recognition by PpiC is likely phosphorylation independent, although a different specificity may be conferred by the β3–β4 loop in PpiC (Fig. 4C). Consistent with a phosphorylation‐independent mechanism, the electrostatic potential surface at the putative catalytic site of PpiC is fully negative (Fig. 4D), a finding that suggests a specificity for positively charged residues preceding proline.

### Dimerisation and swapping

**Fig. 5:**
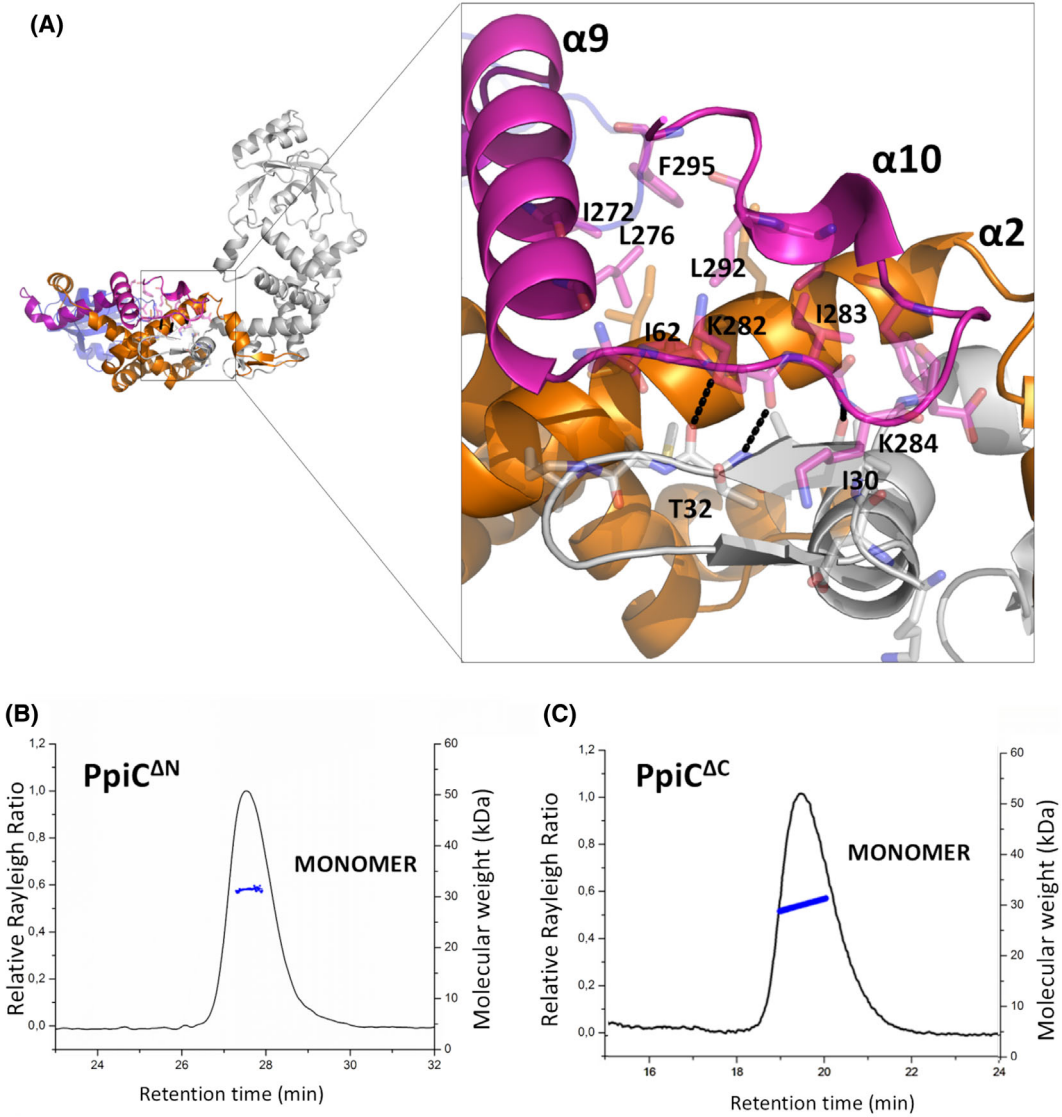
The role of *N*‐ and *C*‐termini in PpiC dimerisation. (A) Cartoon representation of PpiC structure. The inset shows a zoom of the interface between the two protomers in the PpiC dimer. Key residues in protomer–protomer interactions are drawn in sticks. (B) Analytical size exclusion chromatography coupled with light scattering (SEC‐LS) of PpiC^ΔN^ and (C) of PpiC^ΔC^. Relative Rayleigh ratios (left scale) and derived molar masses (right scale, blue points) are plotted versus elution time. Panel A was generated using pymol1.1.

The crystal structure of PpiC, similar to its homologous structures, suggests that an important contribution to protein dimerisation is given by protein‐swapping. Domain‐swapping is a widespread structural mechanism that favours protein oligomerisation in several different contexts. Indeed, proteins able to assemble through domain‐swapping are capable of reaching functional properties that are not available to their monomeric counterparts [52, 53]. In the PpiC crystal structure, swapping is stabilised by multiple backbone–backbone (e.g. I30‐K284 and T32‐K282), backbone–sidechain (K34‐A279 and G35‐N68) and sidechain–sidechain (e.g. N53‐N53) hydrogen‐bonding interactions (Fig. 5A). Also, a compact hydrophobic core is formed by the small *C*‐terminal helix α10 (residues 290–295) and the side chains of Ile272, Leu276, and Ile283 (Fig. 5A). To better understand molecular determinants of the PpiC dimerisation process, we recombinantly produced two truncated forms where either the *N*‐terminal swapping region (1–50) or the *C*‐terminal region 291–336 were depleted. These two truncated forms, here PpiC^ΔN^ and PpiC^ΔC^, were analysed for their structural features in solution.

Light‐scattering analysis of PpiC^ΔN^ shows a single peak, with an average MW of 31.2 ± 0.4 kDa, corresponding to a monomeric form of the protein (Fig. 5B). This finding confirms a strong engagement of the swapped *N*‐terminal β‐hairpin‐helix motif (residues 29–50) in the dimerisation process. The same analysis shows that PpiC^ΔC^ is also monomeric, with an average MW of 29.3 ± 0.7 kDa (Fig. 5C). Given the flexible nature of the charged *C*‐terminal region 304–336, as confirmed by the absence of electron density for these residues, this result suggests a role of the α10 helix in dimerisation (Fig. 5A). By interacting with α2 and α9 helices of the NC domain, the α10 helix stabilises the conformation of the strand connecting α9 and α10, which is directly interacting with the swapped β‐hairpin of the next chain (Fig. 5A).

**Fig. 6:**
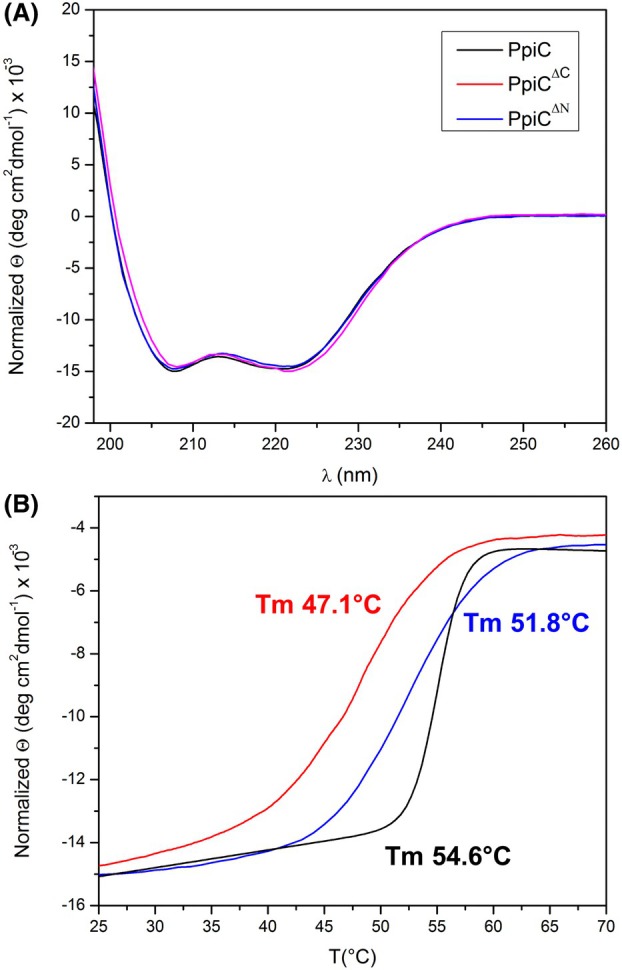
Effect of *N*‐ and *C*‐terminal truncations on PpiC stability. (A) Far Ultraviolet Circular Dichroism (UV‐CD) spectra recorded for PpiC (black), PpiC^ΔN^ (blue) and PpiC^ΔC^ (red) at 25 °C in a phosphate buffer at pH 7. (B) Thermal unfolding curves obtained by following changes in the CD signal at 222 nm as a function of temperature for PpiC, PpiC^ΔN^ and PpiC^ΔC^. Melting temperatures (Tm) of the three PpiC variants are shown in colour code.

Circular dichroism (CD) studies show that neither the *N*‐terminal nor the *C*‐terminal truncation affect the structural integrity of the protein. Indeed, far‐UV CD spectra of PpiC, of PpiC^ΔN^ and PpiC^ΔC^ are characteristic of α‐helical proteins, and all fully superposable (Fig. 6A). However, the truncations affected the protein thermal stabilities. Indeed, thermal denaturation curves, recorded at 222 nm, provided Tm of 55 °C for PpiC, of 52 °C or PpiC^ΔN^ and of 48 °C for PpiC^ΔC^ (Fig. 6A,B). In addition to different values of Tm, thermal denaturation curves show clear differences in the shape of the thermal transition plot, which appears fully cooperative in the case of PpiC, but not for the truncated forms (Fig. 6B). The difference in thermal stabilities, but not in the secondary structures of proteins, suggests that the decreased Tm of PpiC^ΔN^ and of PpiC^ΔC^ may be ascribed to the loss of stabilising interactions provided by dimerisation. Therefore, we decided to further characterise PpiC unfolding thermodynamics using differential scanning calorimetry (DSC).

### Unfolding thermodynamics of PpiC by differential scanning calorimetry

**Fig. 7:**
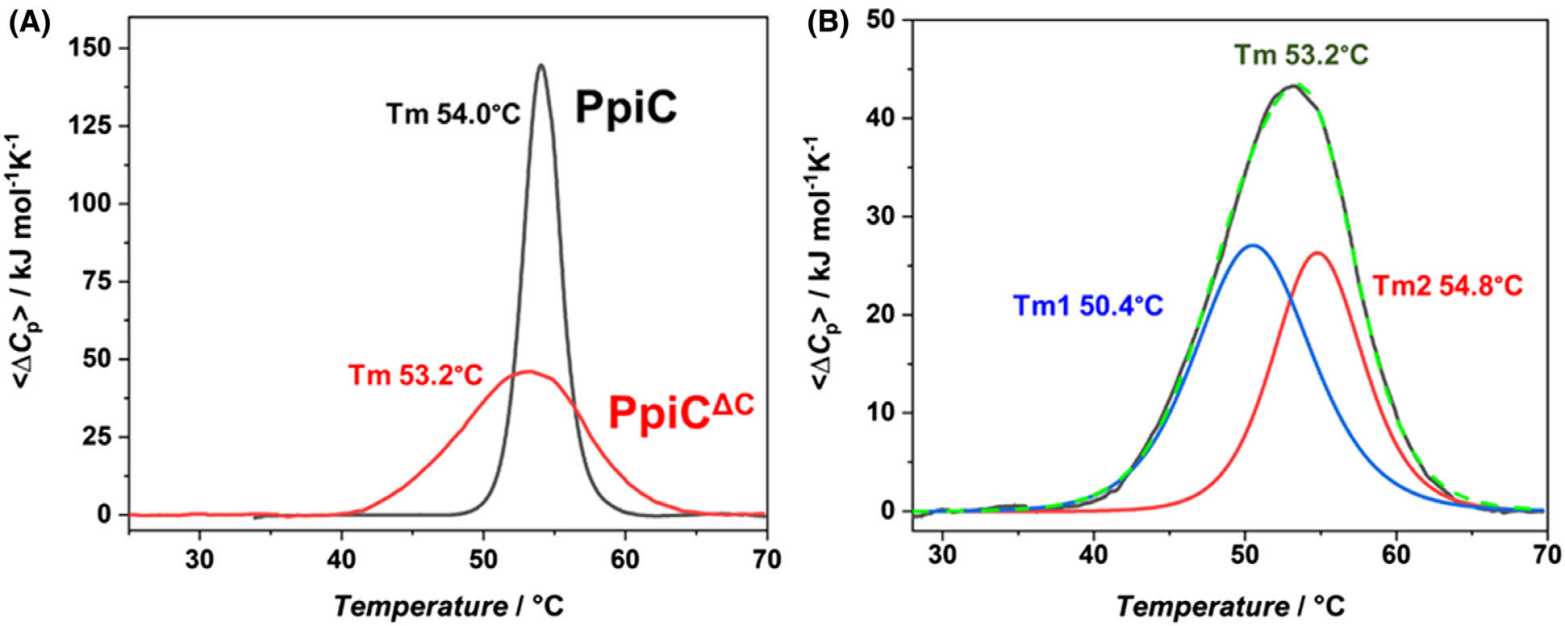
Differential scanning calorimetry (DSC) studies of dimeric and monomeric PpiC. (A) Overlapping of DSC profiles of PpiC (black) and PpiC^ΔC^ (red). Protein concentration was 1 mg·mL^−1^ (27 μm), in PBS buffer, pH 7.4. (B) DSC curves after deconvolution analysis of the calorimetric profile of PpiC^ΔC^. The two deconvoluted peaks and the corresponding melting temperature (Tm) values (Tm1, Tm2) are shown in blue and red, respectively. The sum of the deconvoluted peaks (green) superposes with the experimental profile (dashed black).

DSC was used to characterise the thermodynamics of unfolding of the dimeric PpiC and of the monomeric truncated form PpiC^ΔC^. As shown in Fig. 7A, DSC thermograms recorded at 1.0 mg·mL^−1^ highlight completely different unfolding mechanisms for the two proteins, as indicated by the sharp peak in the PpiC thermogram and the shallow peak in the case of PpiC^ΔC^. The enthalpy of the reaction is directly determined using calorimetry (Δ*H* ^cal^). To analyse the experimental data, we applied the model‐free van't Hoff data analysis to discriminate between different possibilities of protein‐unfolding, for example two‐state unfolding, non‐two state unfolding, and oligomer unfolding of the protein [54]. Indeed, the ratio Δ*H* ^vH^/Δ*H* ^cal^, can suggest different models of unfolding, (a) when Δ*H* ^vH^/Δ*H* ^cal^ = 1, the protein unfolding is well described by a two‐state model (i.e., the protein contains a single energetic domain); (b) if Δ*H* ^vH^/Δ*H* ^cal^ <1, the thermogram indicates that partially overlapping transitions occur and the protein unfolds according to a non‐two‐state model (i.e. the protein contains domains which unfold independently); (c) if Δ*H* ^vH^/Δ*H* ^cal^ > 1, the thermogram indicates a protein‐unfolding coupled to subunit dissociation [54]. Results of model‐free van't Hoff data analysis (Table 4) suggest that PpiC unfolds in a single transition of the dimer N_2_ = 2D with a concomitant unfolding and dimer dissociation, as the enthalpies ratio Δ*H* ^vH^/Δ*H* ^cal^ was 2.9. In a different way, the Δ*H* ^vH^/Δ*H* ^cal^ ratio of 0.6 for PpiC^ΔC^ is indicative of a non‐two‐state model with more overlapping independent transitions (Table 4A). These data suggest in PpiC^ΔC^ that the parvulin domain (holding a PPIase function) and the NC domain (holding a foldase function) unfold independently. Contrarily, unfolding of parvulin and NC domains in the PpiC dimer occurs in a concerted manner, likely due to the stabilising interactions involved in dimerisation (Fig. 5). Further deconvolution of the DSC profile of PpiC^ΔC^ (Fig. 7B) showed best fit using three different curves, corresponding to the unfolding of PpiC^ΔC^ and of its two domains. Tm values and corresponding enthalpies are reported in Table 4B. Altogether, DSC data show that monomer‐monomer interactions in PpiC dimer determine a fully cooperative unfolding of parvulin and NC domains in the PpiC molecule. Differently, the non‐cooperative unfolding of the monomeric PpiC^ΔC^ suggests the loss of those interactions which allow a cross‐talk between parvulin and NC domains.

**Table 4:** Thermodynamic parameters of PpiC and PpiC^ΔC^ unfolding, as measured by DSC.

(a) DSC parameters					
Protein	Tm (°C)	Δ*H* ^cal^ (kJ·mol^−1^)	*C*_p, max_ (kJ·mol^−1^·K^−1^)	Δ*H* ^vH^ (kJ·mol^−1^)	Δ*H* ^vH^/Δ*H* ^cal^
PpiC	54.0 ± 0.2	510 ± 25	143.6	1501	2.9
PpiC^ΔC^	53.2 ± 0.2	508 ± 25	45.9	319	0.6

(b) DSC parameters after the deconvolution analysis of PpiC^ΔC^ thermograms						
Protein	Tm (°C)	Δ*H* ^cal^ (kJ·mol^−1^)	Tm1 (°C)	Δ*H*_1_ (kJ·mol^−1^)	Tm2 (°C)	Δ*H*_2_ (kJ·mol^−1^)
PpiC^ΔC^	53.2 ± 0.2	509 ± 25	50.4 ± 0.1	260 ± 3.6	54.8 ± 0.05	230 ± 3.8

## Discussion

Peptidyl‐Prolyl Isomerases (PPIases) play a vital role in various cellular functions, as they accelerate the cis‐trans interconversion of the peptide bond before proline, a process that is intrinsically slow and plays an essential role in protein‐folding [55]. The PPIase family is highly conserved in eukaryotes, bacteria and archaea [56]. The human pathogen *E*. *faecium*, one of the most notorious nosocomial pathogens belonging to the ESKAPE family, encodes for the PPIase PpiC. In previous works, we showed that PpiC is a promising vaccine antigen [33] and that antibodies against PpiC are opsonic *in vitro* and protective *in vivo* also against *S. aureus* [34]. Despite this important role in both the *E*. *faecium* cellular life and as a promising vaccine antigen, this crucial enzyme of *E*. *faecium* has been poorly characterised. To gain a clearer understanding of the biological role of PpiC in *E*. *faecium*, we have solved its X‐ray crystal structure. We show that PpiC adopts a dimeric bowl‐like shape, where two NC domains of the two chains are kept together through the swapping of the *N*‐terminal regions. Based on structural and biophysical data, we aimed to dissect the structural basis of PpiC dimerisation. Indeed, dimerisation is an important feature for the foldase action of PPIases [36]. It was shown for PrsA of *B. subtilis* that, in addition to the PPIase domain, the *N*‐ and *C*‐terminal domains are essential for bacterial function *in vivo* [26, 39], suggesting that the non‐PPIase activity of these domains is required along with the PPIase domain activity. In *L. monocytogenes*, dimerisation of PrsA is also required for virulence [36]. Also, domain‐mapping experiments revealed that the binding between PrsA of *S. aureus* and its client protein SpA specifically required the NC domains but not the PPIase domain, suggesting that the NC domains of PrsA play a role in substrate recognition independent of the PPIase activity [57]. Recently, it was shown that PrsA of *S. aureus* also formed dimers through their NC domains [34, 57].

The crystal structure of PpiC reported here allowed us to identify key interactions for PpiC dimerisation, namely between the *N*‐terminal β‐hairpin regions 20–49 and the *C*‐terminal helix α10 (Fig. 5). Several hydrogen bonds and hydrophobic contacts stabilise the interaction between swapped β‐hairpins and the core of the NC domain. Analysis of protein interface, performed using the software PISA [58], shows that the dimer formation area occurs with through the burial of a small surface. Indeed, the Accessible Surface Area (ASA) of the PpiC dimer is 35332.3 Å^2^ whereas the Buried Surface (BSA) is 1656.1 Å^2^. To dissect important interactions for the dimer formation, we generated truncated variants lacking either the *N*‐ terminal β‐hairpin‐helix motif (PpiC^ΔN^) or the *C*‐terminal helix α10 (PpiC^ΔC^). We observed that both PpiC truncated forms, different in the way that the variant corresponded to the mature wild type protein, were unable to dimerise. Moreover, we observed different unfolding mechanisms of monomeric PpiC^ΔN^ and PpiC^ΔC^ variants compared to the native enzyme, as measured using CD spectroscopy and differential scanning calorimetry. The dimerisation of PpiC is indeed associated with a high cooperativity of unfolding, consistent with the multiple hydrophobic interactions mediated by the *C*‐terminal helix 296–300 in the dimeric model of the PpiC structure. On the contrary, a scarcely cooperative unfolding process characterises PpiC^ΔC^. This feature elucidates the important role of domain‐swapping in PpiC and other PrsA‐like PPIases. Indeed, domain‐swapping enables the formation of stable oligomers despite the small interface region characterising the dimer formation. The unfolding profile we observe for PpiC suggests that the PpiC biological dimer behaves like a compact monomeric protein, where parvulin and NC domains unfold cooperatively. On the other hand, the small interface region guaranteed by domain‐swapping allows the dimer to expose a large surface to interactions with the client proteins. When comparing the structure of PpiC with that of other PPIases, we observe a large conformational variability, a fundamental property of foldases, which need to adapt to different client proteins. The degree of variability in the orientations between the PPIase (parvulin) and chaperone (NC) domains could suggest that the two domains function independent of each other. However, the cooperativity of unfolding of PpiC, which is lost in its monomeric forms, highlights the existence of an inter‐domain cross‐talk between NC and parvulin domains, likely governed by domain‐swapping. This finding suggests that the functions of the two domains are not independent.

Overall, our work provides a structural basis to the understanding of the role of PPI‐containing foldases, confirming that these proteins act far above catalysing cis‐trans isomerisation and provide an extended flexible albeit well‐structured hydrophobic and hydrophilic surface, helping client proteins to refold on the bacterial membrane. Structural data released here are a prerequisite to the design of advanced vaccine antigens, using a structural vaccinology approach, which has been demonstrated to lead to antigens with stronger immune‐stimulating properties [35, 59, 60]. In addition, our work delivers structural data for the future design of specific PPIase inhibitors as novel antimicrobials against gram + pathogens.

## Materials and methods

### Cloning strategy of PpiC and its truncated versions PpiC^ΔN^ and PpiC^ΔC^

Plasmid pQE30, containing the gene‐encoding for the protein mature PpiC (residues 25–336) from *E*. *faecium* and a six His‐tag at the *N*‐terminus spaced by a GS linker, was obtained from Ludwig Maximillian University of Munich (Germany). The oligonucleotide primers were ordered from Eurofins Genomics Italy S.R.L (Milan, Italy) and used to amplify the nucleotide sequence corresponding to the truncated version PpiC^ΔN^ and PpiC^ΔC^ by Polymerase Chain Reaction (PCR) are reported in Table 5. The amplified fragments were digested with NcoI/XhoI restriction enzymes and then ligated into pETM‐13 plasmid (EMBL, Heidelberg, Germany), enconding for a six histidine tag directly linked to the *C*‐terminus of the protein, digested with the same enzymes (New England BioLabs, Ipswich, MA, USA). Recombinant plasmids were propagated using DH5α *E. coli* competent cells and further purified with a Qiagen mini‐prep kit (Hilden, Germany). Since terminal His‐tags are known to potentially affect the experimental reproducibility [61], we performed all experiments at pH 7.4 to keep histidine residues in an unprotonated form and therefore limit possible bias.

**Table 5:** Sequences of the forward and reverse oligonucleotide primers used to amplify the nucleotide sequence corresponding to the truncated version PpiC^ΔN^ and PpiC^ΔC^.

Construct	Primers	
	Forward	Reverse
PpiC^ΔN^	CATGCCATGGCGCTTGAATCATCGAACCAATCCT	CCGCTCGAGTTTTGATGAATCAGTTGTTTCTTC
PpiC^ΔC^	CATGCCATGGATACTAATAAAGATATCGCAAC	CCGCTCGAGCACATTTTCAAAGGCATCATCT

### Recombinant production of PpiC and its truncated versions PpiC^ΔN^ and PpiC^ΔC^

The recombinant vectors carrying selected genes were introduced into different competent *E. coli* bacteria via the heat shock method. To detect the optimal growth and production conditions, we explored different types of *E. coli* competent cells, isopropyl β‐*d*‐1‐thiogalactopyranoside (IPTG) concentrations and temperatures of induction. Once information on the optimal conditions for expression has been obtained, proteins were expressed on a large scale using *E. coli* BL21 (DE3), using a previously reported protocol [35]. Cultures were kept, with the appropriate antibiotics, at 37 °C with agitation 180 rpm until OD600 reached 0.4–0.6. Subsequently, the bacterial cultures were induced with optimal concentration of IPTG (0.8 mm for PpiC and 0.2 mm for PpiC^ΔN^ and PpiC^ΔC^) and transferred to 22 °C o/n with agitation 180 rpm for protein production.

For harvesting, cultures were centrifuged for 20 min at 4 °C, at 7000 rpm. Pellets were dissolved in a lysis buffer (300 mm NaCl, 50 mm Tris–HCl, 2.5% glycerol, pH 7.8) containing protease inhibitor cocktail (Roche, Basel, Switzerland) and further sonicated on ice for 15 min (10‐s sonication followed by 10‐s rest). Then, the solutions were centrifuged at 4 °C for 45 min, at 16 000 rpm. The supernatants were transferred onto a gravity‐flow column loaded with Ni‐NTA resine (Qiagen, Hilden, Germany), where washing steps were performed with buffer A (300 mm NaCl, 50 mm Tris–HCl, 2.5% glycerol, pH 7.8 and 10 mm imidazole). Elution of the proteins was performed at a final concentration of 150 mm imidazole and concentrated on Amicon 10 kDa cut‐off (Merck Millipore, Burlington, MA, USA). Proteins were further purified on Superdex 200 increase 10/300 GL (GE Healthcare, Chicago, IL, USA) (PpiC) or Superdex 75 increase 10/300 GL (GE Healthcare) (PpiC^ΔN^ and PpiC^ΔC^) pre‐equilibrated in a buffer containing 50 mm Tris–HCl, NaCl 150 mm, and 5% glycerol, pH 7.8. When the yield of the protein was high, columns with higher capacities were used [16/600 Superdex 200 and 75 pg (GE Healthcare)]. Proteins were concentrated on Amicon 10 kDa cut‐off (Merck Millipore).

### Circular dichroism studies

For the analysis of the conformational state of proteins, far‐UV circular dichroism (CD) spectra were registered using a Jasco J‐810 spectropolarimeter with a Peltier temperature control system (model PTC‐423‐S; Jasco Europe, Cremella (LC), Italy). Far‐UV measurements at protein concentration of 0.2 mg·mL^−1^ were carried out in a 20 mm phosphate buffer, at different temperatures, using a 0.1 cm optical path length cell. The range of the reported wavelengths 198–260 was fine‐tuned as a function of the observed HT voltage. The spectra, recorded with a time constant of 4 s, a 2 nm bandwidth, and a scan rate of 10 nm·min^−1^, were signal averaged over at least two scans. The final spectra were expressed as molar ellipticity [*θ*] (deg cm^2^·dmol^−1^) per residue. The temperature of the transition midpoint (*T*_m_) was investigated by monitoring the change in ellipticity at 222 nm, while increasing the temperature from 25 to 70 °C with the scan rate of 1.0 °C per minute. The reversibility of the transition was checked by lowering the temperature to 25 °C and re‐scanning the sample. The mean residue ellipticity, [*θ*] in deg·cm^2^·dmol^−1^, was calculated from the equation:

$$\left[\theta \right]=\left[\theta \right]\mathrm{obs}\cdot \mathrm{mrw}\cdot {\left(10\cdot l\cdot C\right)}^{-1}$$

where [*θ*]obs is the ellipticity measured in degrees, mrw is the mean residue molecular mass (Da), *C* is the protein concentration in g·l^−1^and l is the optical path length of the cell in cm.

### Differential scanning calorimetry

DSC measurements were performed by means of a high sensitivity Nano DSC (TA Instruments, New Castle, DE, USA) equipped with 300 μL twin gold capillary cells, pressurised to 3 atm. The partial molar heat capacity of the protein in solution was measured as a function of temperature and the excess heat capacity function (<Δ*C*_p_>) was obtained after baseline subtraction. Buffer‐buffer scans were recorded under the same conditions and subtracted from sample profiles. The experiments were performed at protein concentration of 1 mg·mL^−1^ (27 μm), in PBS buffer at pH 7.4. A scan rate of 1.0 °C·min^−1^ in the temperature range of 25–80 °C was chosen for all the experiments. Data analysis was performed by using the nano analyse software, provided by the manufacturer and plotted using the Origin software package (OriginLab, Northampton, MA, USA).

The van't Hoff denaturation enthalpy, Δ*H* ^vH^, was calculated according to the following equation [62]:

$$\Delta H^{\mathrm{vH}}=\frac{\mathrm{nR}T_{m}^{2}C_{p,\max}}{\Delta H^{\mathrm{cal}}}$$

where *n* is 6 or 4 for a bimolecular or monomolecular process, respectively; *T*_m_ is the midpoint of the unfolding curve, *C*_p, max_ is the maximal molar excess heat capacity measured at *T*_m_ and Δ*H* ^cal^ is the molar enthalpy calculated by integrating the area under the transition peak. *R* is the gas constant (*R* = 8.314 J·mol^−1^·K^−1^).

### Light‐scattering

Multiple Angle Light‐Scattering Experiments on purified proteins were analysed by size‐exclusion chromatography coupled to a DAWN MALS instrument (Wyatt Technology, Santa Barbara, CA, USA) and an Optilab rEX (Wyatt Technology). Five hundred micrograms of each sample were loaded on Superdex 200 increase 10/300 GL (GE Healthcare) (PpiC) or Superdex 75 increase 10/300 GL (GE Healthcare) (PpiC^ΔC^) equilibrated in a buffer containing 50 mm Tris–HCl, NaCl 150 mm, and 5% glycerol, pH 7.8. A constant flow rate of 0.5 mL·min^−1^ was applied. The on‐line measurement of the intensity of the Rayleigh scattering as a function of the angle as well as the differential refractive index of the eluting peak in SEC was used to determine the weight average molar mass (Mw) of eluted protein, using the astra 5.3.4.14 (Wyatt Technologies) software.

### Molecular modelling

The search model used for Molecular Replacement was obtained using alphafold3.0 [41]. The reliability of the AF predictions was assessed both by the Local Distance Difference Test (LDDT) score, a per‐residue confidence score, with values greater than 90 indicating high confidence, and values below 50 indicating low confidence. We considered only parts of the model with a plDDT value higher than 70 to be reliable.

### Crystallisation of PpiC, data collection and refinement

Purified PpiC protein in 150 mm NaCl, 50 mm Tris/HCl, pH 7.8, was concentrated to 20 mg·mL^−1^. Crystallisation trials were conducted at 20 °C using the hanging drop vapour‐diffusion method as described elsewhere [63]. Crystals were obtained after 1–2 days. The reservoir solution contained 0.1 M Sodium acetate trihydrate pH 4.6, 0.1 M Cadmium chloride hydrate, 30% v/v Polyethylene glycol 400. Diffraction data were collected at the ESRF (Grenoble, France) at 100 K and at the wavelength 0.8731 Å. Cryoprotection of the crystals was achieved by rapid soaking in a solution consisting of 0.095 M Cadmium chloride hydrate, 0.095 M Sodium acetate trihydrate pH 4.6, 28.5% v/v Polyethylene glycol 400, 5% (vol/vol) glycerol. Diffraction images were processed using HKL3000 [64].

The crystal structure of PpiC was determined using molecular replacement and the program Phaser [40]. The best search model was obtained through modelling, using alphafold3.0 [41]. The refinement started with data up to 3.0 Å resolution and increased to the highest resolution in successive manual and automated rounds of refinement using the software coot [65] and phenix [66], respectively. The software pymol was used to visualise the structure and generate figs [67].

## Author contributions

RB planned experiments; VN, EK, OG and FS performed experiments; RB, FRS, JH and PDV analysed data; RB wrote the original draft; all authors revised the draft.

## Conflict of Interest

The authors declare no conflict of interest.

## Peer review

The peer review history for this article is available at https://www.webofscience.com/api/gateway/wos/peer‐review/10.1111/febs.70160.

## Data Availability

All the plasmids and relevant data are available upon request from the corresponding authors. The model of monomeric PpiC used for Molecular Replacement was deposited to ModelArchive (https://www.modelarchive.org/doi/10.5452/ma‐eg3yv/). The coordinates and structure factors have been submitted to the Protein Data Bank under the accession code 9IBN.
